# Adsorbed Conformations of PCE Superplasticizers in Cement Pore Solution Unraveled by Molecular Dynamics Simulations

**DOI:** 10.1038/s41598-017-16048-3

**Published:** 2017-11-30

**Authors:** Tsuyoshi Hirata, Jun Ye, Paulo Branicio, Jianwei Zheng, Alex Lange, Johann Plank, Michael Sullivan

**Affiliations:** 1 Nippon Shokubai Co., Ltd., Research Division, Suita, 564-0034 Japan; 2 Institute of High Performance Computing, Materials Science and Engineering, Connexis, 138632 Singapore; 3 University of Southern California, Mork Family Department of Chemical Engineering & Materials Science, Los Angeles, CA 90089-0241 USA; 4 Technische Universität München, Chair for Construction Chemistry, Garching, D-85747 Germany

## Abstract

The conformations of polycarboxylate ether (PCE) type superplasticizer polymers adsorbed on the surface of MgO in cement pore solution are simulated by molecular dynamics (MD). Three types of PCEs commonly applied to concrete are simulated, namely a methacrylate type PCE (PCEM-*P*), an allyl ether type PCE (PCEA-*P*), and an isoprenyl ether type PCE (PCEI-*P*) with ethylene oxide (EO) unit numbers (*P*) of 25, 34 and 25, respectively. It is observed that the adsorbed layer thickness is inversely proportional to the experimentally measured adsorbed amount at the initial paste flow of 26 ± 0.5 cm. Simulation results indicate that the adsorbed layer thickness is sensitive to the initial polymer orientations against the model MgO surface. I.e., polymer molecules initially placed parallel/perpendicularly against the MgO surface gradually forms a train shaped or a loop and tail adsorption profile, respectively. As a result, the loop and tail shaped conformation gives a higher layer thickness.

## Introduction

Polycarboxylate ether-based superplasticizers (PCEs) adsorb onto the cement surface electrostatically with their negatively charged carboxylic acids in a polymer backbone while the polyethyleneglycol (PEG) side chains stretch toward the water phase, resulting in an excellent cement-dispersing effect^1^.

PEG molecules are surrounded by a large layer of structured water resulting from hydrogen bonds^2,3^. Accordingly, PEG side chains of PCEs that adsorb on cement would turn the surface of cement particles hydrophilic, making the agglomerated cement particles to be dispersed by steric repulsion of the PEG chains. For instance, it is well known that PEG stabilizes hydrophobic colloid acting as a protective agent^4–6^. Later, de Gennes^7,8^ established quantitatively that the steric repulsion generated by the grafted chains correlates with the adsorbed layer thickness (ALT) of the adsorbed polymer. It has been demonstrated by a variety of work that the steric repulsion of PCEs increases with increasing ALT^1,9–15^. There is very roughly linear trend between the ALT value and the PEG side chain length or the polymer’s hydrodynamic radius.

However, the PCE polymer structures used for these early studies were limited and sometimes unknown. The PCE dosage varies for different types of PEG macromonomers used at the preparation of a mortar or concrete with the settled initial fluidity. The reasons for that remain unsolved. Therefore, understanding the correlation of a PCE molecular structure with the cement dispersing effect would promote the development of more efficient superplasticizers to enhance concrete performance.

Recently, molecular dynamics (MD) simulations have been applied to the PCEs to elucidate its working mechanism^16–20^. However, the molecular size of these model PCEs was much smaller than those typically used for industrial applications in order to reduce computation cost, furthermore, only pure water was used, or only Na^+^, Ca^2+^ and Cl^−^ were additionally added to mimic a cement pore solution.

In a previous work, we simulated the polymer conformations of the PCEs with the actual *Mw* used in experiments^21^. We considered different polymer backbones in water and in a synthetic cement pore solution. The PCEs used were common types utilized in the concrete industry, namely a methoxy polyethyleneglycol mono methacrylate–sodium methacrylate copolymer (PCEM-*P*), a methoxy polyethyleneglycol mono allyl ether–sodium maleate copolymer (PCEA-*P*) and a polyethyleneglycol mono (3-methyl-3-butenyl) ether–sodium acrylate copolymer (PCEI-*P*) with ethylene oxide (EO) unit numbers (*P*) of 25, 34 and 25 respectively. PCEA is an alternating copolymer formed from allyl ether and maleic anhydride monomers which alternate along the polymer backbone. In contrast, PCEM is a random copolymer^22^ while PCEI is a block copolymer with ABA, ABAA and AA blocks as evidenced by^13^C NMR spectroscopy^23^, where A and B refer to the monomer units containing the carboxylic acid group and PEG side chains, respectively.

It was found that the backbones of those PCEs are considerably stretched in water due to electrostatic repulsion between negatively charged carboxylic acid groups, whereas in synthetic cement pore solution the backbones are significantly distorted and shrunken due to the aggregation of the hydrophobic PEGs as well as the salting-out effect on the backbone^21^.

In this paper, we follow this preliminary effort and use MD simulations to investigate the conformations of the same three PCEs polymers adsorbed on an MgO surface in a synthetic cement pore solution. In addition, we also performed mini slump tests and compared results of simulated adsorbed polymer conformations against experiment to gain insights into possible correlation.

Schematically, this paper is organized as follows: First, our results are reported and discussed regarding the working mechanism of the PCE samples, and our findings are compared with those from works of other groups previously published. Then, we give a short conclusion. Lastly, we present our experimental and simulation methodology.

## Results and Discussion

### Dispersing performance of PCEs

To obtain an initial cement paste flow of 26 ± 0.5 cm, the PCE dosages measured are 1.35 mg/g (PCEM-25), 1.40 mg/g (PCEA-34) and 1.00 mg/g (PCEI-25). This result indicates that PCE polymer PCEI-25 has the highest dispersing ability among all the polymers tested.

### PCE adsorption on cement

**Figure 1:**
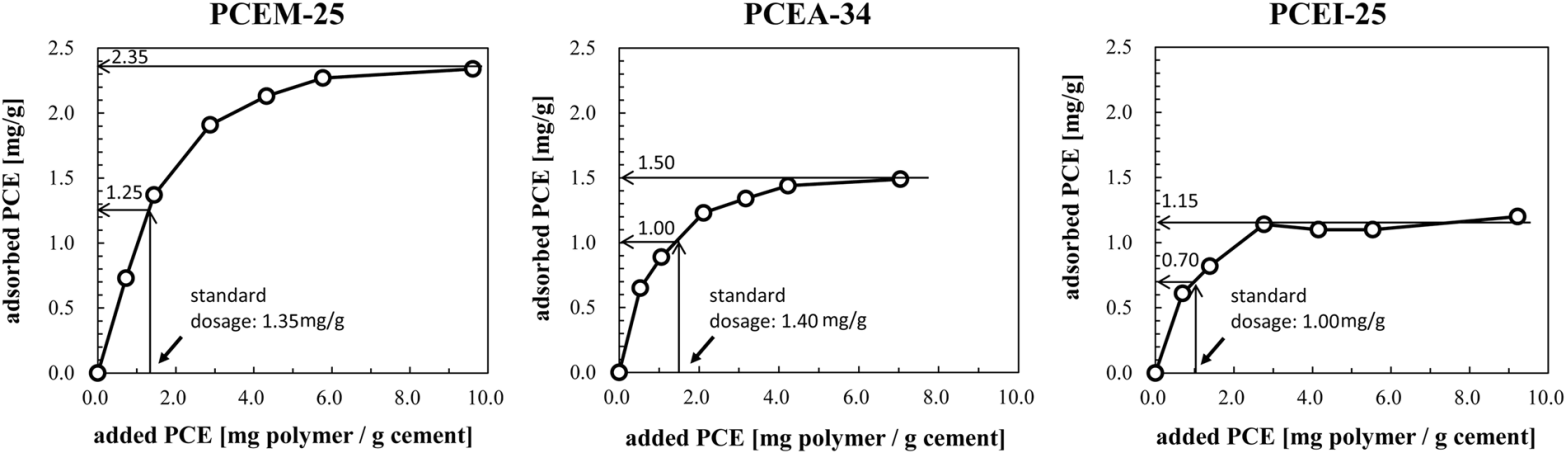
Adsorption isotherms of the three PCE samples on cement.

As exhibited in Fig. 1, the adsorption isotherms have a Langmuir shape, which was in line with those previously reported for monolayer adsorption between polar solutes and substrates^24,25^. The adsorbed amount increases sharply at low dosage, then reaches a plateau where PCE covers all surface by a monolayer. Such adsorption isotherms have also been reported earlier for PCEs^26^. In which saturated adsorption amounts are found as: 2.35 mg/g (PCEM-25) > 1.50 mg/g (PCEA-34) > 1.15 mg/g (PCEI-25). In contrast, the adsorbed amounts at the dosage required for a 26 ± 0.5 cm cement paste spread value (hereinafter, “the standard adsorbed amounts”) are 1.25 mg/g (PCEM-25) > 1.00 mg/g (PCEA-34) > 0.70 mg/g (PCEI-25), indicating that full coverage of the cement surface is not needed and coverage with 53–67% is enough to obtain the targeted flow. The difference in the standard adsorbed amounts of the PCEs seems to suggest the existence of different polymer conformations during adsorption. Later we will discuss about it on the basis of the adsorbed polymer conformation on MgO surface.

### Estimation of the proton density on the MgO surface by zeta potential and MD simulation of short PCE polymer chains

**Figure 2:**
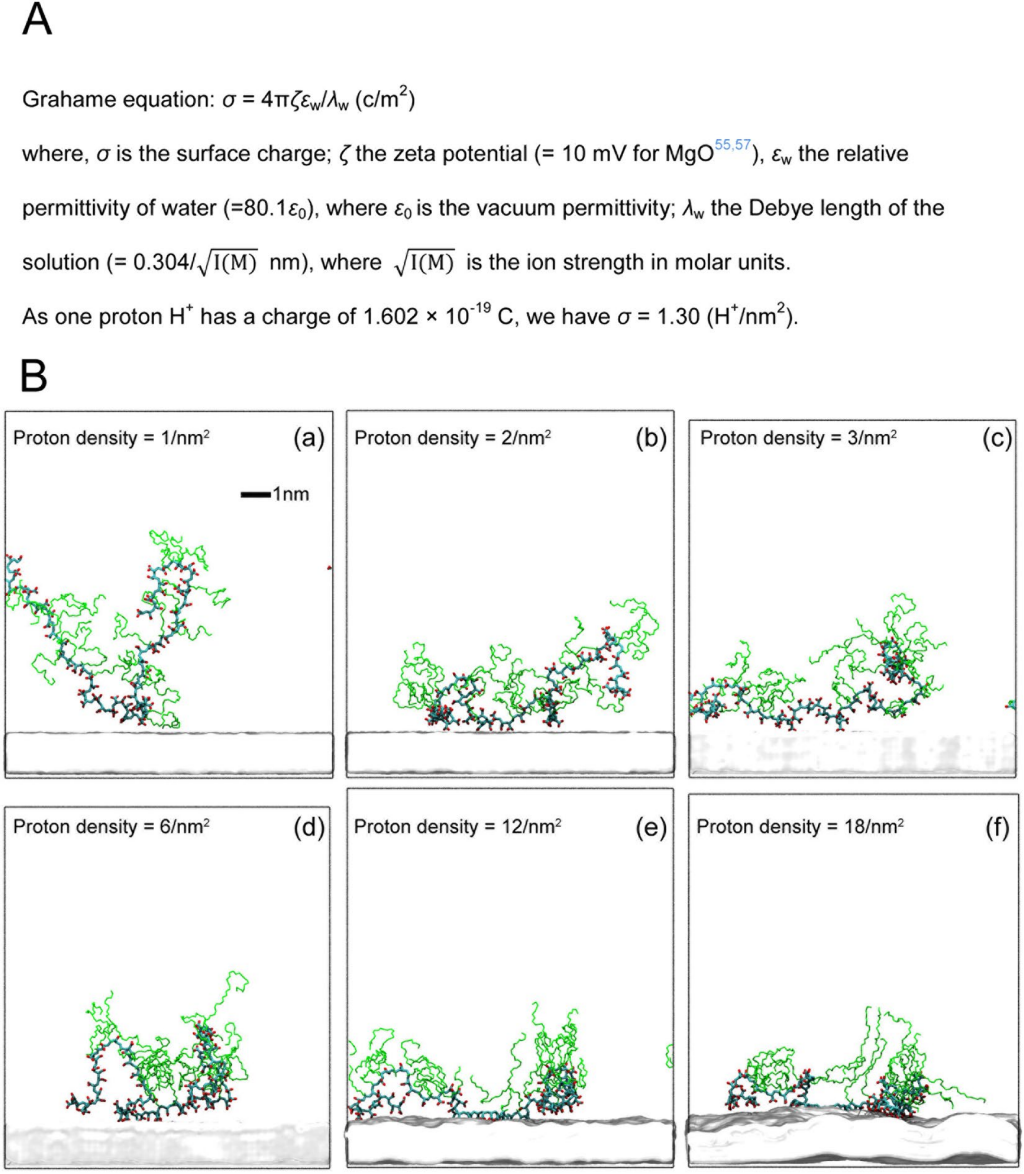
Estimation of the proton density on the MgO surface in cement pore solution. (**A**) Calculation based on the zeta potential of MgO and the use of the Grahame equation. (**B**) Final conformation of PCEI-10 adsorbed on MgO surface for different proton densities (proton/nm^2^): (a) 1, (b) 2, (c) 3, (d) 6, (e) 12 and (f) 18, and their ALT (nm) are: (a) 7.3, (b) 4.4, (c) 5.0, (d) 5.2, (e) 4.1 and (f) 3.6.

The positive charge density (proton density) present on the MgO surface in cement pore solution is estimated based on the experimental data for the zeta potential of MgO and the use of the Grahame equation, indicating that 1 to 2 atoms in about 25 atoms/nm^2^ on MgO surface should be protonated (Fig. 2A). However, the real value of the proton density on the MgO surface is considered to be much larger because the Zeta potential is defined at the slipping plane and does not indicate the actual surface charge. Accordingly, in order to evaluate the best proton density using a modest computational time, a scaled down test is performed in synthetic cement pore solution using PCEI-10, which has a short side chain length of *P* = 10, lower *M*_w_ of 18,100 and a grafting molar ratio τ = 20 mol%. A three layer MgO slab with the surface protonated in the wide range between 1 to 18 protons/nm^2^ is used. MD simulations are performed for 150 ns until relaxed polymer configurations in synthetic cement pore solution are obtained. Successively, the PCEI-10 molecule is placed at the centre of a system with an MgO surface and the adsorption is investigated by running additional 10–20 ns. The results are exhibited in Fig. 2B with the ALT values that are obtained by averaging the values from last 20% of each MD trajectory. It was observed that once the backbone was adsorbed on the surface, the resulting adsorption sites adhere to the surface and they are never desorbed. The ALT values demonstrate that the surface with 12 and 18 protons/nm^2^ makes a PCE backbone to adsorb very intensively (4.1 nm and 3.6 nm), resulting in a completely flat conformation of the backbone, with no observed mobility. The simulations also indicate that a strong adsorption is able to deform the MgO surface, slightly warping it. On the contrary, a density of 1 proton/nm^2^ generates a partial attraction of the PCEI-10 molecule (7.3 nm). As a result, the moderate proton densities of 2, 3, and 6 protons/nm^2^ produce stable layer thicknesses of 4.4, 5.0 and 5.2 nm, respectively. Therefore, we have selected 6 protons/nm^2^ throughout the result of studies for PCEs with doubled chain length because that value offers a balance between a fast adsorption process and preservation of the expected polymer features in the final adsorbed conformations, such as loops and tails.

### Adsorbed conformation on the MgO surface in synthetic cement pore solution by MD simulations

The simulations of polymer conformation in synthetic cement pore solution are carried out until the values of calculated properties indicate a steady state with oscillation around a given value, due to thermal fluctuations. In the previous work^21^, we have demonstrated that whole PCE polymers require MD simulations as long as 200–350 ns and the PEG side chains 10–100 ns to reach a steady state until the initial energy is minimized and the thermal and pressure equilibrations are achieved. The final confirmation of PCEs show generation of PEG clusters, which arguably occurs due to the hydrophobic association of PEGs (i.e. salting-out effect) which is induced in the presence of inorganic ions or high *pH* values^27^. PEG molecules are known to easily aggregate with each other by hydrogen bonds^28,29^. Relaxed PCE backbones are significantly distorted and shrunken due to the salting-out effect as well.

**Figure 3:**
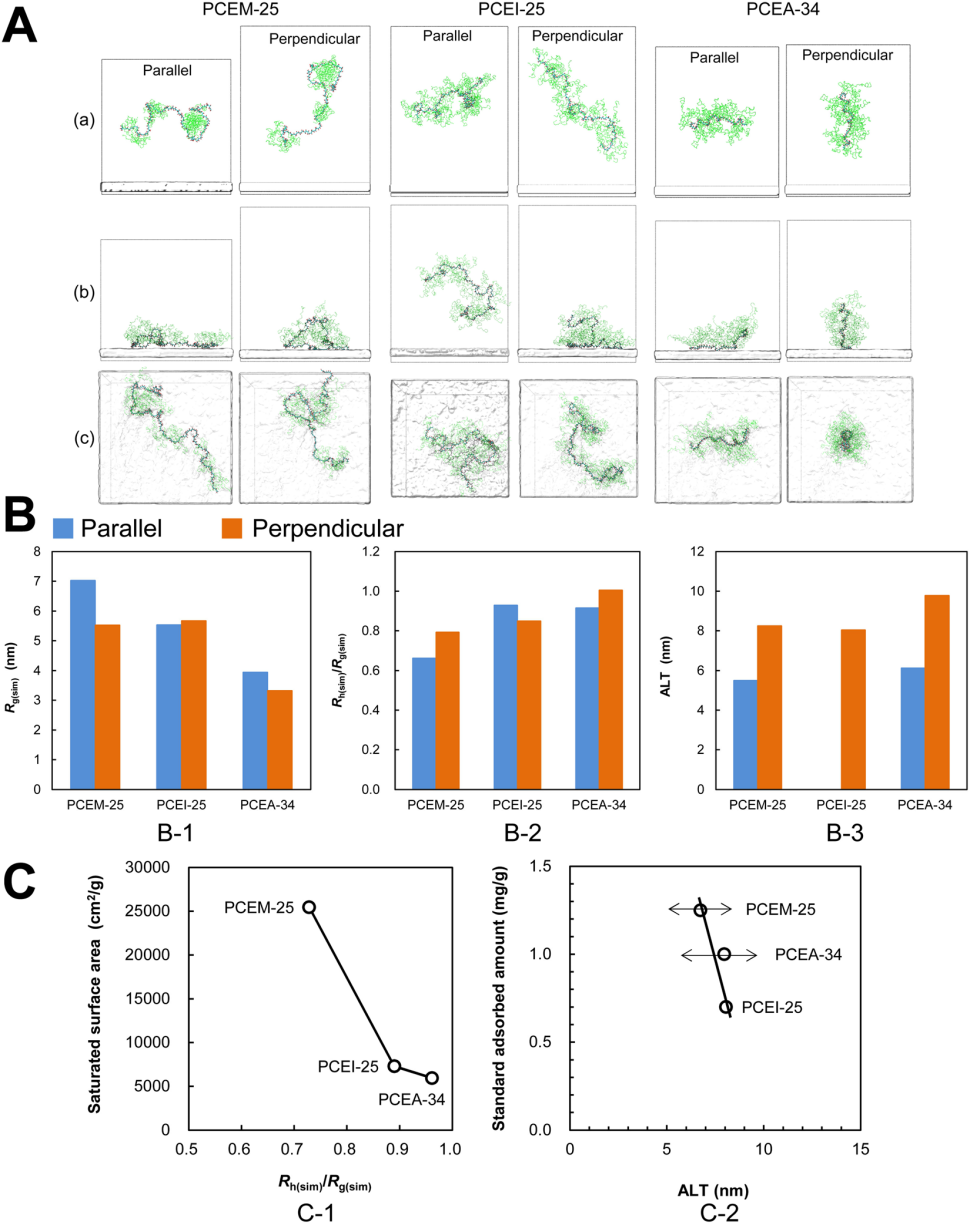
Adsorption properties predicted by simulations for initial parallel and perpendicular orientations. (**A**): Adsorbed conformation of the PCE samples. (a) Initial structure; (b) after 250 ns of dynamic evolution viewed from side and (c) viewed from top. (**B**): Adsorption properties. (**B-1**) *R*_g(sim)_, (**B-2**) The *R*_h(sim)_/*R*_g(sim)_ ratio, (**B-3**) ALT, (**C**): (**C-1**) Relationship between the saturated surface area and the *R*_h(sim)_/*R*_g(sim)_ ratio that is averaged parallel one and perpendicular one. The saturated surface area is calculated by π*R* ^2^_g(sim)_ × {*Ad*_saturated_/(1000 × *M*_w_)} × (*N*_A_/10^14^), where *Ad*_saturated_ is the saturated adsorption amount presented in Fig. 1, *N*_A_ is the Avogadro constant. (**C-2**) Standard adsorbed amount *vs*. predicted ALT.

Successively, the PCE molecules that had been stabilized in cement pore solution are placed at a location with their centre-of-mass coordinates about 10 nm apart from the MgO surface in two distinct orientations: one parallel and one perpendicular to the MgO surface. Figure 3A illustrates: (a) the initial structures of PCEM-25, PCEA-34, and PCEI-25 that are stabilized in synthetic cement pore solution mentioned above; (b) the final conformations of those samples on MgO surface after MD simulations of about 250 ns; and (c) the surface view of the final conformations, respectively. The simulation time of 250 ns for adsorption is sufficient to allow all properties to be converged to steady state values. All the PCE samples adsorbed on MgO surface other than PCEI-25 that is placed in parallel position. The reason is discussed later.

The adsorption conformation depends on the orientation that a stabilized PCE molecule is placed around the centre of the systems. Namely, the parallel placement against the MgO surface introduces adsorption conformation with PCE shape close to train while the perpendicular placement offers adsorption conformations pertaining loop or tail, leading to higher layer thicknesses. Figure 3B shows the adsorption properties of radii of gyration (*R*_g(sim)_), the hydrodynamic radii (*R*_h(sim)_)/*R*_g(sim)_ ratio and the ALT values calculated from the simulations.

The saturated surface areas that are calculated from PCEA-34 and PCEI-25 adsorption simulation results (6000 and 7300 cm^2^/g, respectively) are about two times or more than the specific surface area of 3299 cm^2^/g. It is consistent with a previous report^30^ that the surface area of cement increases by 2 to 2.5 times larger just after water contact. However, PCEM-25 covers about eight times the specific surface area. That can be explained in terms of the *R*_h(sim)_/R_g(sim)_ ratio (Fig. 3C-1). This ratio acts as an indicator of polymer conformation^31^, suggesting that the adsorbed conformation of PCEM-25, PCEI-25 and PCEA-34 changes from closer to linear to that closer to spherical in this order. Accordingly, the linear shaped PCEM-25 molecule can be considered to overlap the *R*_g_ region of neighbouring molecules in solution in contrast with the spherical ones like PCEA-34 or PCEI-25 molecules.

It is assumed that ALT values more than 6 nm are required for superior superplasticizers such as PCEM, PCEA and PCEI (Fig. 3B-3) displaying a lower standard dosage that is required to obtain the initial paste flow of 26 ± 0.5 cm. Figure 3C-2 shows that ALT is inversely proportional, as expected, to the standard adsorbed amount at the initial paste flow of 26 ± 0.5 cm. Here, the ALT values are obtained by averaging the heights for the initial parallel and perpendicular placement simulations. However, the difference of the ALT is only 1.2 nm between PCEM-25 (the lowest one) and PCEI-25 (the highest one), and only 0.1 nm between PCEA-34 and PCEI-25. Here, it should be noted that, due to the high computational cost, only one ALT value is calculated for each orientation. The results show that, the deviation of the ALT in each PCE is comparatively large. Therefore, additional simulations should be performed to provide statistically meaningful values. Ideally, random initial orientations of molecule should be considered as well as different sequence of monomers for PCEI-25 and PCEM-25.

In addition, it is probably reasonable to assume that other factors beyond the ALT may affect the dispersion of the cement particles. Indeed, previous reports have deduced, for instance, that the non-adsorbed PCE supplements the cement-dispersing effect of the adsorbed PCE, acting as lubricants^32,33^ and that the hydrophilic PCE molecule shows superior effect of cement-dispersing to hydrophobic one^34^. The influence of adsorbed PCE molecules on the morphology of the hydrated grains may be considered as well.

**Figure 4:**
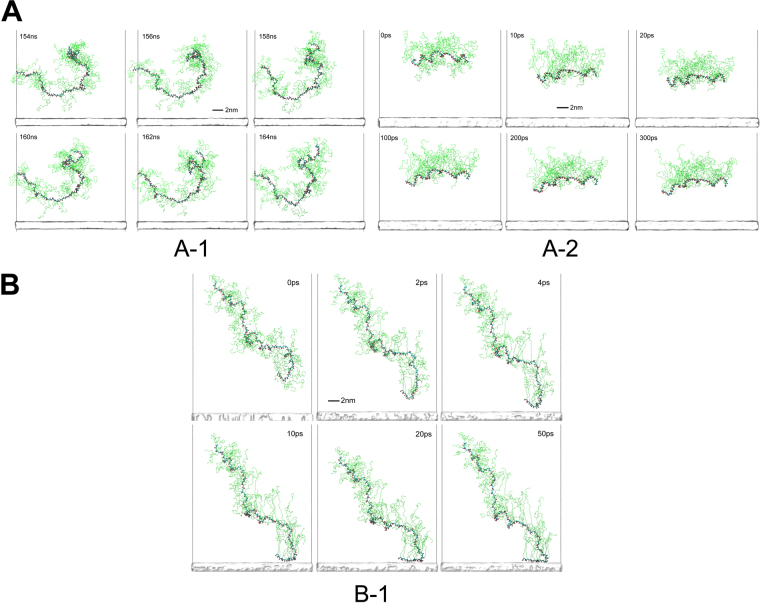
Snapshots of side views of PCEs samples adsorbing conformation (with (**A**) parallel initial orientation and (**B**) perpendicular initial orientation) on MgO surface with proton density of 6/nm^2^. PEG side chains in PCEs are colored in green to gain better contrast against the backbone atoms. (**A-1**) PCEI-25. The snapshots are taken from MD simulations from 154 to 164 ns of a trajectory with total length of 250 ns. (**A-2**) PCEA-34. The snapshots are taken from initial stage of MD simulations from 0 to 300 ps of a trajectory with total length of 250 ns. (**B-1**) PCEI-25. The snapshots are taken from initial stage of MD simulations from 0 to 50 ps of a trajectory with total length of 250 ns.

We should discuss the reason why PCEI-25 is not adsorbed on MgO surface when it is placed at a parallel initial position. At first, it is conceived that the carboxylic acid groups on a PCEI-25 backbone do not work as the adsorption sites because of a perfect coverage with the PEG side chains, but PCEA-34 with a larger grafting ratio adsorbs. Hence, the conformation change of PCEI-25 and PCEA-34 has been carefully compared at the parallel placement. The simulation results are shown in Fig. 4A-1 (PCEI-25) and A-2 (PCEA-34). In Fig. 4A-1, we can see many stretched PEG chains are pointing to the MgO surface during a full trajectory of 200 ns where PCEI-25 backbone is about 3 nm away from the MgO surface. These PEG side chains can induce steric hindrance effect to prevent further downwards movement of the PCE backbone. Therefore, these stretched PEG side chains play somehow “shielding” role owing to such steric hindrance effect. In comparison, PCEA-34 adsorbs by pulling the aggregated PEG lump onto the backbone probably due to an easy rotation of maleic acid moiety toward a positively charged MgO surface even at the initial stage (Fig. 4A-2). Such conformation is obviously beneficial to the further adsorption of PCE backbone onto the MgO surface. In contrast, when a PCEI-25 molecule is placed at a perpendicular position (Fig. 4B-1), it can adsorb on MgO surface through the carboxylic acids at the end of the backbone. Those carboxylic acids less covered with PEG side chains can easily attack a positively charged MgO surface.

Many researchers obtained ALT values within 1–5 nm, which are smaller than the values in this paper, for PCEM type superplasticizers by the atomic force microscope (AFM) measurements in a KCl, NaCl or synthetic cement pore solution^9–11,35^. The ALT values predicted by the mean-field model^9,36,37^ are 3.0 nm, 3.1 nm and 4.0 nm for PCEM-25, PCEI-25 and PCEA-34, respectively. However, a general concern has been expressed about values for ALT obtained by AFM measurements as the AFM tip might compress the adsorbed polymer and thus lead to ALT lower than the actual values^32^, which would explain the contrast with our direct values obtained from analysis of atomic positions from simulations. In addition, the mean-field model disregards the aggregation of PEG side chains, which our simulation has shown to be significant. Indeed, Sakai *et al*.^14^ showed the significantly large layer thickness of 4.54–11.51 nm in allyl ether−maleic acid−styrene copolymer by measuring the amount of water passing through an alumina filter with minute holes of 100–200 nm in which PCE molecules are adsorbed.

## Conclusions

The adsorption behaviour of three types of common PCE concrete superplasticizers (PCEM, PCEA and PCEI) on MgO surface in synthetic cement pore solution was studied by full atomistic MD simulations. Results indicate that polymer molecules placed initially parallel or perpendicularly against an MgO surface leads to adsorption in shape closer train or that pertaining loop and tail, respectively. A loop and tail shape produces a higher layer thickness. The predicted layer thickness is inversely proportional to the amount of adsorbed polymer at the initial paste flow. A PCE with the adsorbed layer thickness higher than 6 nm is a necessary condition for an excellent superplasticizer such as PCEM, PCEA and PCEI with a lower standard dosage. Nonetheless, it is suggested that other factors beyond the adsorbed layer thickness should be taken into consideration as well.

## Materials

### Cement

The cement used in this study was an ordinary Portland cement (CEM I 52.5 N Milke^®^classic from Heidelberg Cement, Geseke plant, Germany). Its phase composition as determined by quantitative X-ray diffraction (Bruker D8 advance instrument, software Topas 4.0) is presented in Table 1. The specific surface area of 3299 cm^2^/g is measured using a Blaine instrument (Toni Technik, Berlin/Germany). An average particle size (*d*_50_ value) of 12.02 µm is determined by laser granulometry CILAS 1064 (Cilas, Marseille, France), and the density measured by a Helium pycnometer (Quantachrome, Odelzhausen, Germany) is 3.16 g/cm^3^.

**Table 1:** Phase composition of CEM I 52.5N sample^a^.

Phase	wt.%
C_3_S, m	70.1
C_2_S, m	11.0
C_3_A, c	5.1
C_3_A, o	2.1
C_4_AF, o	2.5
Free lime (Franke)	0.9
Periclase (MgO)	0.0
Anhydrite	2.5
Hemihydrate^b^	0.3
Dihydrate^b^	0.4
Calcite	3.3
Quartz	0.7

^a^Determined by Q-X-ray diffraction analysis using Rietveld refinement.

^b^Determined by thermogravimetry.

### PCE copolymers

**Figure 5:**
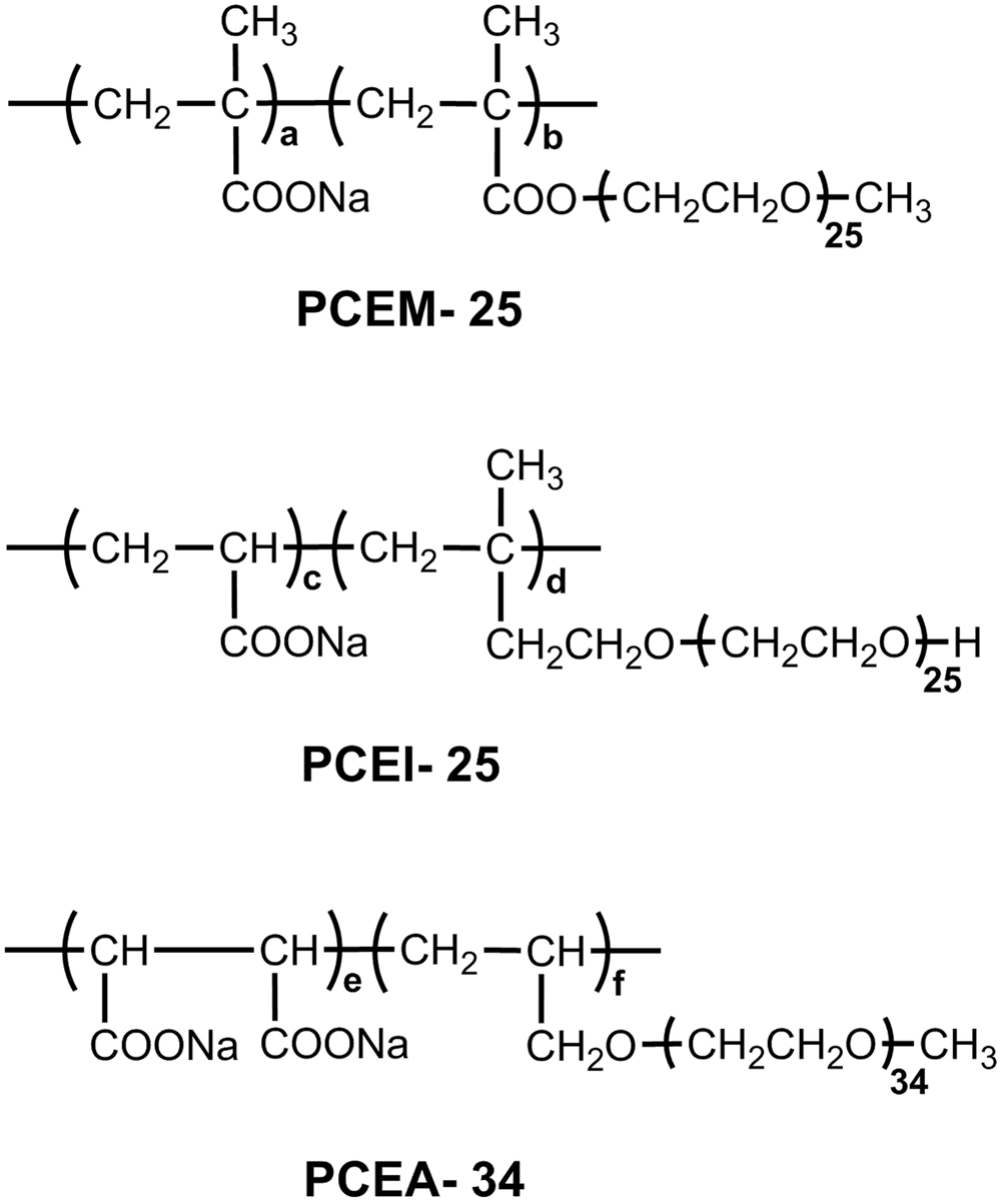
Chemical structures of the PCE samples studied. It is reproduced from authors’ own work^21^ published with ICE Publishing.

The chemical structure of the PCE samples investigated, PCEM-25, PCEI-25, and PCEA-34 are shown in Fig. 5. These structures are the same as those used in our previous work^21^. Their individual synthesis methods have been described previously^38–40^. The properties of the synthesized polymers are summarized in Table 2.

**Table 2:** Monomer composition and analytical data of the PCE samples.

PCE-P^a^	*M*_w_^b^	T^c^: mol %	Composition: wt%^d^		Polymer content^b^: %	Polydispersity index, PDI (Mw/Mn)
			PEG monomer	Carboxylic monomer		
PCEM-25	68,800	21	75	25	96.1	2.2
PCEI-25	93,800	31	85	15	92.2	2.6
PCEA-34	63,100	50	91	9	70.4	2.8

^a^ *P*: number of ethylene oxide (EO) units.

^b^Determined by SEC.

^c^Grafting molar ratio.

^d^Weight ratio of monomers used in the polymerization process.

The *M*_*w*_, the polymer content (monomer conversion) and the polydispersity index (PDI) were determined by size exclusion chromatography (SEC, Waters Alliance 2695 from Waters, Eschborn, Germany) equipped with refractive index (RI) detector 2414 (Waters, Eschborn, Germany) and a three-angle dynamic light-scattering detector (mini Dawn from Wyatt Technologies, Santa Barbara, CA, USA): prior to application on the columns, the polymer solutions were filtered through a 0.2 μm filter. The polymers were separated on an Ultrahydrogel precolumn and three Ultrahydrogel (120, 250 and 500) columns (Waters, Eschborn, Germany) using 0.1 M aqueous sodium nitrate (NaNO_3_) solution (adjusted to pH 12.0 with sodium hydroxide (NaOH)) as an eluent at a flow rate of 1.0 mL/min. The value of refraction index increment (d*n*/d*c*) used to calculate *M*_*w*_ and *M*_*n*_ for all polymers is 0.135 mL/g (value for polyethylene oxide)^41^.

The SEC spectra of the PCE samples are shown in our previous work^21^. The polymer content (percentage of monomers converted to polymer) was determined by the area (%) of the polymer profile in the RI diagram.

The initial structure of the PCEs before geometric optimization is also shown in our previous work^21^. All polymer models are constructed using the polymer builder function of the Materials Studio package. It is emphasized that the PCEA side chains line up much more densely along the backbone than in PCEM and PCEI because of the alternating copolymer structure.

In all three PCE samples, PCEM-25, PCEI-25 and PCEA-34, the weight ratio of PEG macromonomer/carboxylic monomer is optimized for best dispersion. For example, the weight ratio of the monomers in PCEM-25 was 75/25 (PEG monomer/acid monomer) which is in good agreement with the value which has been suggested in previous work^42^. In this paper, the dosages applied in the cement paste tests are all converted to the values for the pure polymers without residual monomers to compare the effects of the polymers themselves.

### Experimental and simulation procedures

#### Performance test with cement using ‘mini slump’ test

For the determination of the paste flow, a ‘mini slump’ test was utilised and carried out as follows. First, a constant water-to-cement (*w/c*) ratio of 0.3 was chosen. At this *w/c* ratio, the dosages of the polymers required to reach a spread of 26 ± 0.5 cm are determined. Generally, the polymer is dissolved in the required amount of mixing water placed in a porcelain cup. The amount of water contained in the polymer solution is subtracted from the amount of mixing water. Next, within 5 s, 350 g of cement are added to the mixing water and agitated manually for 1 min, then rested for 1 min without stirring. This is followed by intensive stirring for another 2 min. The cement paste is immediately poured into a Vicat cone (height 40 mm, top diameter 70 mm, bottom diameter 80 mm), then placed on a glass plate and the cone is vertically lifted. The resulting spread of the paste is measured twice, the second measurement being at a 90° angle to the first one, and averaged to give the spread value. All tests were carried out at 22 °C.

#### Adsorption measurement

The PCE adsorption on cement is measured using the depletion method. The non-adsorbed portion of polymer remaining in solution at equilibrium condition is determined by analyzing the total organic carbon (TOC) content of the solution. In a typical experiment, 50 g of cement, 15 g of water and the amount of PCE to be tested are filled into a 50 mL centrifuge tube, shaken in a wobbler (VWR International, Darmstadt/Germany) for 4 minutes at 2400 rpm and then centrifuged for 15 minutes at 8500 rpm. The supernatant is diluted with deionized water. The TOC content of the solution is determined by combustion at 890 °C on a High TOC II instrument (Elementar Analysensysteme, Hanau/Germany). From the difference between the TOC content of the polymer reference sample and the TOC content of the supernatant, the adsorbed amount of PCE is calculated.

#### Molecular Dynamics Simulations

MD simulations are performed using the GROMACS package^43–47^ version 5.1.2. Simulations are performed using the NVT canonical ensemble (constant temperature, volume, and number of atoms) at room temperature (300 K) with a time step of 2 fs. The LINCS algorithm^48^ and its parallel version P‐LINCS^49^ are applied to constrain bonds involving light atoms such as hydrogen. The visual molecular dynamics (VMD) visualization package^50^ is used to the analyze radius of gyration and hydrodynamic radius for the whole polymer based on MD trajectories saved every 5000 steps (10 ps) and averaged in the last 30 ns of the simulations. Prior to each NVT run, geometry optimization and a 5 ns MD relaxation process under the isothermal-isobaric ensemble (NPT) with pressure P = 1 bar and temperature (300 K) is applied.

The duration of each NVT run is adjusted based on the convergence of the properties. For the models considered that is expected to be from 150 to 500 ns. For the adsorbed models, ALT is calculated and used as a convergence criterion.

#### Model considerations

Initial conformations of PCEs for adsorption study in this work are obtained from those relaxed conformations in our previous work^21^. The previous simulations on the PCE conformations in solution^21^ are performed using the standard MD procedure using a periodic simulation box. However, the simulations of adsorption require slab geometry with 2D *xy* periodic boundary conditions and an artificial confinement in the *z* direction (perpendicular to the slab surface). Here, we use a confinement of the system within a *z* layer and Gromacs special features for this kind of simulation. Those features included wall setup scheme, *xy* periodic boundary conditions, and the use of the Ewald-zfac method to minimize unphysical interactions of atoms with periodic images in the *z* direction.

PCEs mainly adsorb on positively charged hydrates of cement phases such as C_3_A, C_4_AF and especially on ettringite crystallizing on hydrating cement grains^51–53^. However, the cement surface is unclear due to the hydrates changing with hydration time, with an inherent complexity^54^. Therefore, inert MgO is used as a model representing a complex cement particle^10,11,55–57^.

The synthetic cement pore solution used in the simulations is generated using the following amount of salts: 1.72 g/l gypsum (CaSO_4_ 2H_2_O), 6.959 g/l sodium sulfate (Na_2_SO_4_), 4.757 g/l potassium sulfate (K_2_SO_4_), and 7.12 g/l potassium hydroxide (KOH) (pH = 13.1). The salts are assumed to be completely dissociated in water. Based on this assumption the exact molar concentrations of Ca^2+^, SO_4_^2−^, Na^+^, K^+^, and OH^−^ are calculated to be: Ca^2+^: 0.010 M, Na^+^: 0.098 M, K^+^: 0.181 M, SO_4_^2−^: 0.086 M and OH^−^: 0.127 M. For simulation purposes, the molar concentrations are converted to volumetric concentrations: Ca^2+^: 6.087 × 10^−3^/nm^3^, Na^+^: 5.901 × 10^−2^/nm^3^, K^+^: 0.109/nm^3^, SO_4_^2−^: 5.203 × 10^−2^/nm^3^, and OH^−^: 7.642 × 10^−2^/nm^3^.

#### Force Field Model for Polymer, MgO and ions

The ‘optimised potentials for liquid simulations’ (OPLS) all atom^58–62^ force field is employed to describe the interatomic forces for the model of all PCE polymers, the MgO substrate, and ions including Ca^2+^, Na^+^, K^+^, Cl^−^. Force field parameters for OH^−^ and SO_4_^2−^ are taken from the ‘chemistry at Harvard macromolecular mechanics’ (CHARMM) force field^63^.

## Electronic supplementary material

Supplementary Information
