# Supplementary material for: Adsorbed Conformations of PCE Superplasticizers in Cement Pore Solution Unraveled by Molecular Dynamics Simulations
Source: Sci Rep. 2017 Nov 30;7:16599. doi: 10.1038/s41598-017-16048-3 (PMC5709394; doi:10.1038/s41598-017-16048-3)
Supplement: Supplementary file 1 — Supplementary Information [file 41598_2017_16048_MOESM1_ESM.pdf]

Supplementary Information

Article Title: Adsorbed Conformations of PCE Superplasticizers in Cement Pore Solution Unraveled by Molecular Dynamics Simulations

Authors: Tsuyoshi Hirata[1*], Jun Ye[2], Paulo Branicio[3], Jianwei Zheng[2], Alex Lange[4], Johann Plank[4], Michael Sullivan[2]

[1]Nippon Shokubai Co., Ltd., Research Division, Suita, 564-0034, Japan

[2]Institute of High Performance Computing, Materials Science and Engineering, Connexis, 138632

[3]University of Southern California, Mork Family Department of Chemical Engineering & Materials Science, Los Angeles, CA 90089-0241, USA

[4]Technische Universität München, Chair for Construction Chemistry, Garching, D-85747, Germany

*Corresponding author, Tsuyoshi Hirata, Tel: +81-6-6317-2825; Fax: +81-6-6317-2992; E-mail address: tsuyoshi_hirata@shokubai.co.jp

Figure S1 to S3 are time evolution of various calculated properties of PCEs investigated during adsorption processes. Each figure corresponds to the cases listed in Figure 3 A of the main text.

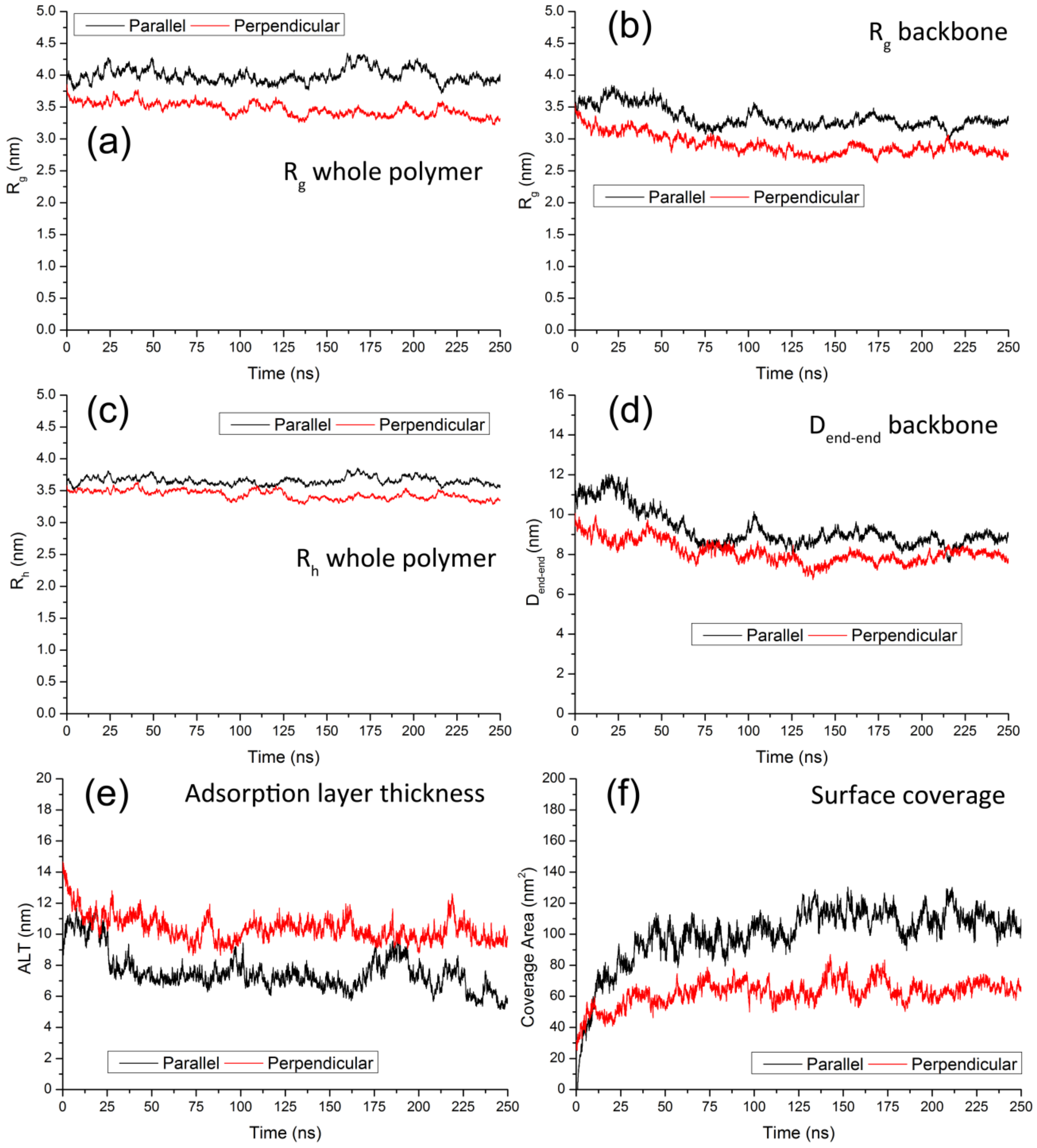

Figure S1. Time evolution of calculated quantities for PCEA-34 with parallel and perpendicular initial orientation adsorbed on MgO surface with proton density of $6/nm^2$.

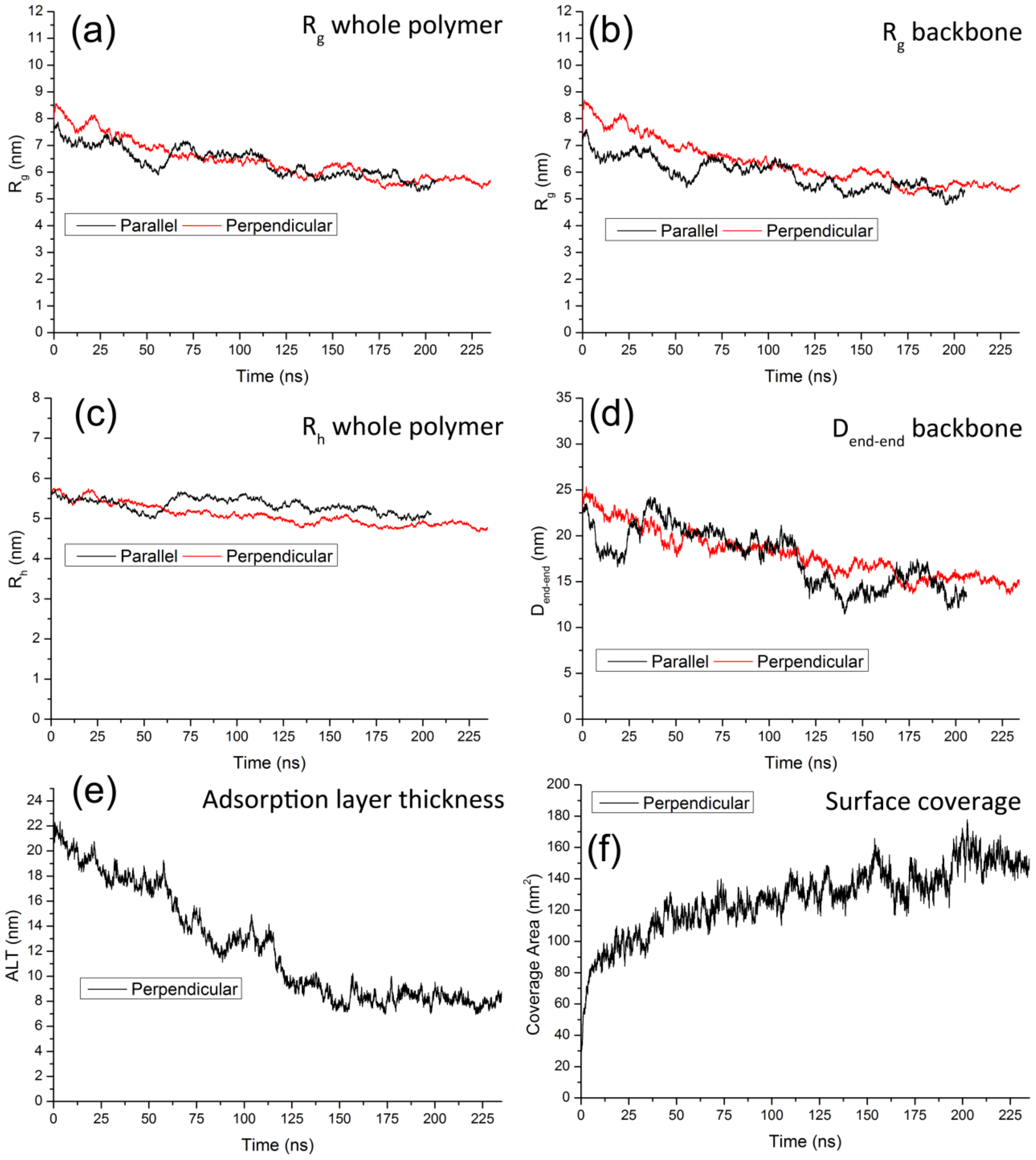

Figure S2. Time evolution of calculated quantities for PCEI-25 with parallel and perpendicular initial orientation adsorbed on MgO surface with proton density of 6/nm^2.

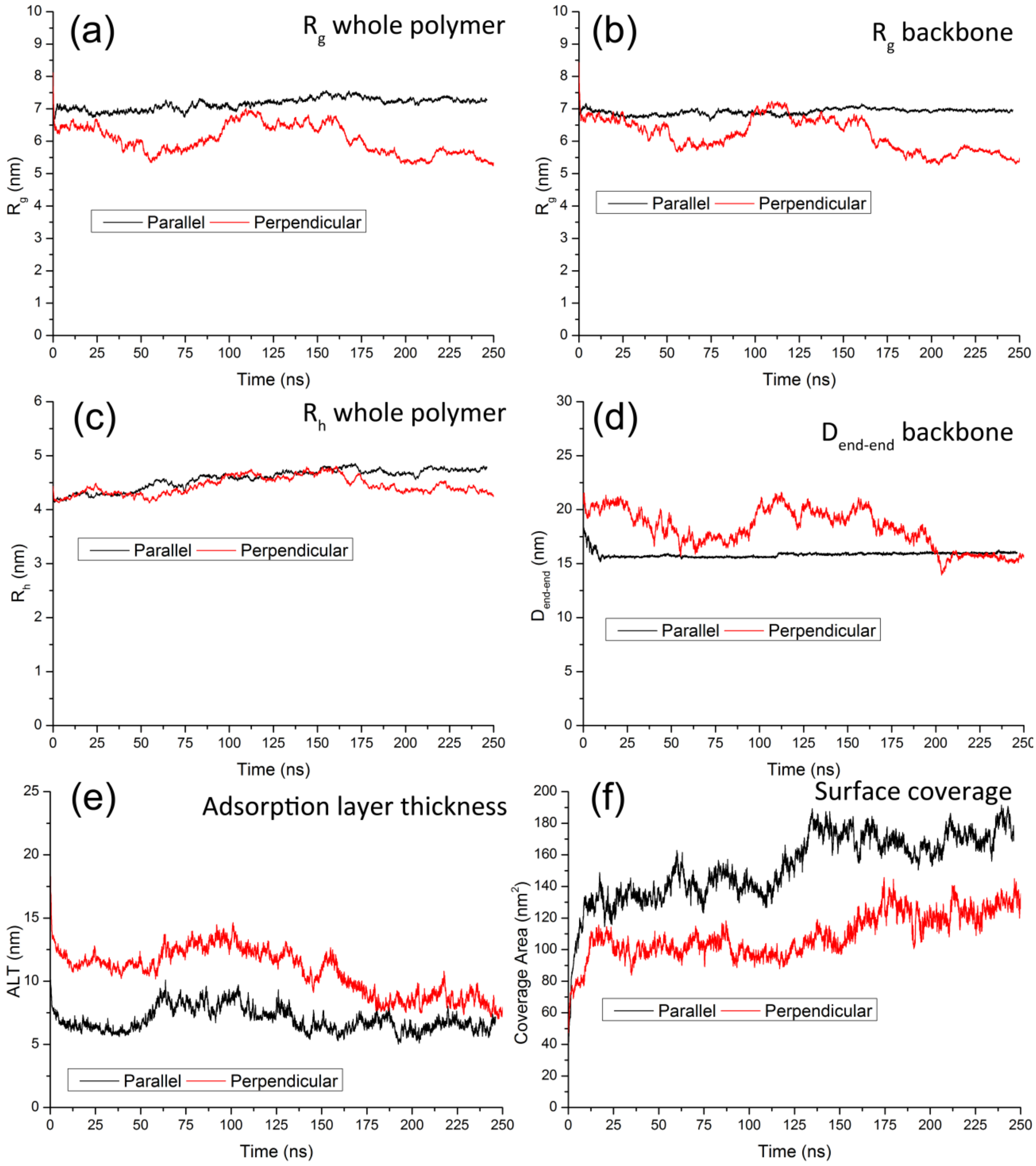

Figure S3. Time evolution of calculated quantities for PCEM-25 with parallel and perpendicular initial orientation adsorbed on MgO surface with proton density of 6/nm^2.
